# Wellbore Stability through Novel Catechol-Chitosan Biopolymer Encapsulator-Based Drilling Mud

**DOI:** 10.3390/gels8050307

**Published:** 2022-05-16

**Authors:** Zhichuan Tang, Zhengsong Qiu, Hanyi Zhong, Yujie Kang, Baoyu Guo

**Affiliations:** 1 School of Petroleum Engineering, China University of Petroleum (East China), No. 66 Changjiang West Road, Economic & Technical Development Zone, Qingdao 266580, China; b17020063@s.upc.edu.cn (Z.T.); zhonghanyi@126.com (H.Z.); 17854210608@163.com (Y.K.); 2 Drilling Technology Research Institute, Shengli Petroleum Engineering Corporation Limited of SINOPEC, Donying 257064, China; guobaoyu719.slyt@sinopic.com

**Keywords:** wellbore stability, biopolymer, encapsulator, water-based drilling fluids, chemical strengthening

## Abstract

The problem of wellbore stability has a marked impact on oil and gas exploration and development in the process of drilling. Marine mussel proteins can adhere and encapsulate firmly on deep-water rocks, providing inspiration for solving borehole stability problem and this ability comes from catechol groups. In this paper, a novel biopolymer was synthesized with chitosan and catechol (named “SDGB”) by Schiff base-reduction reaction, was developed as an encapsulator in water-based drilling fluids (WBDF). In addition, the chemical enhancing wellbore stability performance of different encapsulators were investigated and compared. The results showed that there were aromatic ring structure, amines, and catechol groups in catechol-chitosan biopolymer molecule. The high shale recovery rate demonstrated its strong shale inhibition performance. The rock treated by catechol-chitosan biopolymer had higher tension shear strength and uniaxial compression strength than others, which indicates that it can effectively strengthen the rock and bind loose minerals in micro-pore and micro-fracture of rock samples. The rheological and filtration property of the WBDF containing catechol-chitosan biopolymer is stable before and after 130 °C/16 h hot rolling, demonstrating its good compatibility with other WBDF agents. Moreover, SDGB could chelate with metal ions, forming a stable covalent bond, which plays an important role in adhesiveness, inhibition, and blockage.

## 1. Introduction

The problem of wellbore stability in oil and gas drilling engineering has always been a worldwide technical problem. Especially in the complex deep-seated, deep-sea oil and gas, special structural wells, unconventional reservoirs, and other drilling projects, the problem of borehole wall instability is more prominent. According to incomplete statistics, the cost of dealing with borehole instability in drilling construction accounts for 50% of the cost of drilling fluid [1,2,3].

Hydration of shale is a complex process. Shale has low permeability and clay minerals such as montmorillonite are contained in the pores [4]. Na-montmorillonite consists of negatively charged clay sheets, which are similar to pyrophyllite layers, whose negative charge is compensated by interlayer sodium ions to maintain electrical neutrality [5]. The surface hydration first occurs after the montmorillonite meets water. At this stage, the expansion pressure generated by hydration is large and the volume expansion is small. With the hydration, the expansion pressure decreases rapidly [6].

The surface hydration causes crystallization expansion. Ionic hydration begins when the surface is hydrated [7]. Ion hydration brings hydration film to clay and hydration ions compete with water molecules for the connection position of crystal plane of clay. After surface hydration and ionic hydration of clay minerals such as sodium montmorillonite, the hydrated ions dissociate in the liquid away from the surface of clay minerals, forming a diffusion double layer between clay minerals [8]. The combined action of double layer repulsion and osmotic pressure produces hydration, that is, osmotic hydration. The volume of clay minerals expands under this action. At this stage, 1 g montmorillonite can absorb 10 cm of water. The volume increases by nearly 20 times, and the crystal layer spacing increases to 13 μm [9].

Therefore, under the huge drilling pressure difference, drilling fluid filtrate is very easy to invade the formation along the micro-porous and micro-fissures of shale [10]. The micro-voids and fractures of shale are rich in clay minerals such as montmorillonite. As sodium ions are very liable to occur in the above-mentioned hydration process in wet environment [11], the lattice of montmorillonite is very liable to cause interlayer crystal distance expansion after meeting water, which is manifested as macroscopic hydration expansion and dispersion. Once shale meets water, it will expand and generate huge expansion force, which will directly endanger wellbore integrity. At the same time, water intruding into shale crevices will also cause shale micro-cracks to expand and extend, resulting in wall instability [12,13].

In recent years, researchers have developed a series of additives which can improve the borehole pressure bearing capacity [14,15,16,17]. In terms of physical plugging, micro-nano particles are mainly used for direct plugging at present. There are many micro-nano pores in shale formation. The size of conventional plugging agent particles is too large to enter into the micro-nano pore in shale, which is easy to accumulate on the surface and cannot form effective plugging [18,19]. At present, nano-particles such as nano-silica, nano-magnesium oxide, iron oxide, zinc oxide, and graphene have been directly used or surface modified and used as plugging agent in the field of drilling fluids [20,21,22,23]. However, due to the high activity of nanoparticles, they are easy to agglomerate and lose their characteristics. At the same time, inorganic nanoparticles have a high rigidity and are easy to disengage and migrate after entering formation pore throat, which is not conducive to plugging [24,25].

In terms of chemical inhibition, different kinds of shale inhibitors have been developed, including inorganic salts, organic salts, macromolecular polymers, low molecular organic amines, polyalcohols, and so on [26,27,28,29]. These inhibitors mainly inhibit hydration expansion and dispersion of shale by restraining hydration of clay surface, encapsulating shale particles, changing wettability of shale surface, and controlling water activity of drilling fluids [28,29,30,31]. With the development of science and technology, the main applications in water-based drilling fluids are mainly silicates, organosilicons, polyalcohols, aluminum-based, and asphalts. With the changes in temperature, pH, salinity, or cloud point effect after they enter the formation, precipitation or insoluble substances are generated to plug the micro-pore and micro-fracture of shale, and then the purpose of sealing and consolidating the borehole wall is achieved. R. Schlemmer [32,33,34] of M-I Drilling Fluid Company has successfully developed an encapsulator to replace sugars and acrylates. At 4% addition, the 8-h expansion rate of shale can be reduced from 73.3% to 15.6%, which has been successfully applied in the Beihai Sea. 5high performance silicate-based muds were chosen by M. Albooyeh [35] out of 81 different formulations of drilling fluids, which showed good rheology properties and all of them were thermally stable. The five performance silicate-based muds lost less than 10 mL before and after hot roll at 160 C/16 h, and the rolling recovery of shale cuttings also increased from less than 20% to nearly 80%. Qi Chu [36] developed a An organosilicon quadripolymer of acrylamide (AM), 2-acrylamido-2-methyl-1-propane sulfonic acid (AMPS), *N*-vinylpyrrolidone (NVP) and a kind of organosilicon monomer by solution free radical polymerization. The test result showed that the organosilicon quadripolymer drilling fluid performance was better than corresponding terpolymer without organosilicon group and shows favorable inhibitive property. EI-Monier [37] developed an environmentally friendly inorganic aluminum/aluminum shale inhibitor encapsulator-A through special size and structure design. The agent is a mixture of inorganic aluminum and aluminum in a certain proportion. It can effectively prevent the active clay from absorbing water, and the expansion rate can be reduced by about 90% after 8 h, while the water absorption of sodium montmorillonite can be reduced by 30%. It has low toxicity and can effectively seal formation pore under high temperature and strong acid environment.

However, there are some limitations of the above additives, such as limited bearing capacity of the well eye, poor compatibility of drilling fluid, weak effect of high temperature, and unfriendly to the environment. The results show that wellbore instability of shale formation is the result of both physical and chemical factors [38,39,40]. So, it is very important to develop an encapsulator which takes physical plugging and chemical cementing into account to solve the problem of wellbore instability in shale formation.

Mussel can firmly adsorb to rocks under strong winds and waves in the ocean. Its foot silk protein is a powerful adhesive and has been widely used in biological and medical fields. At the same time, the surface becomes denser and stronger after mussels adhere to rocks, hulls, etc., which also shows the potential of physical consolidation [41,42].

The active ingredient of this mussel adhesive protein is a catechol group. Inspired by this characteristic, catechol was grafted onto the main chain of chitosan by Schiff base-reduction reaction method. Finally, a novel environmentally friendly biopolymer encapsulators in WBDF, named SDGB, was developed, and the structural characterization and performance evaluation are conducted.

## 2. Results and Discussion

### 2.1. Orthogonal Experiment of SDGB

The main factors affecting polymer performance include the monomer ratio of catechol to chitosan, reaction time, initiator concentration, and reaction temperature. Orthogonal tests with three levels and four factor designs are shown in Table 1, and the results are shown in Table 2. The rolling recovery of shale rock chips was evaluated in SDGB aqueous solution. The temperature has the most significant effect on the rolling recovery of shale rock chips. The following optimal conditions were determined by rolling recovery of shale rock chips: catechol to chitosan molar ratio of 1:2, initiator concentration of 7 wt%, reaction time of 10 h, and reaction temperature of 10 °C.

### 2.2. Characterization of SDGB

#### 2.2.1. Fourier Transform Infrared Spectroscopy (FT-IR) Characterization Test

**Figure 1:**
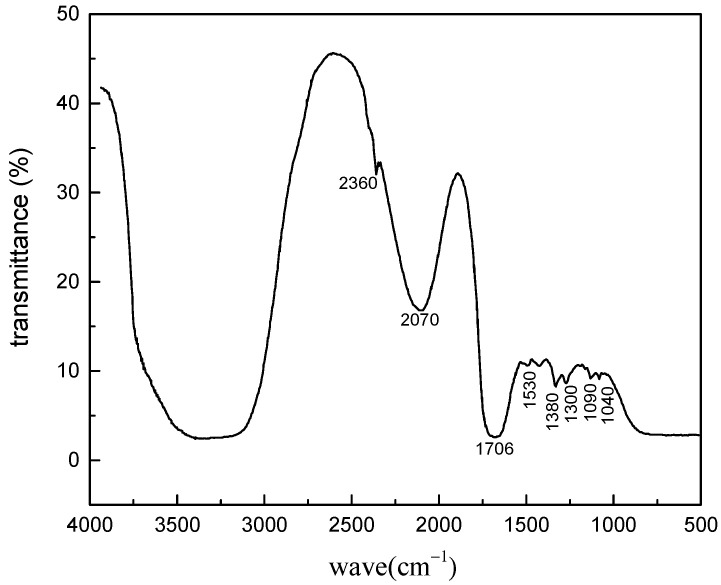
Fourier transform infrared spectrum of SDGB.

The result of FT-IR is shown in Figure 1. It can be seen that the amino group has a stretching vibration absorption peak at 1706 cm^−1^, and the peak at 1380 cm^−1^ belongs to the methyl vibration absorption peak. The typical absorption peaks of O–C stretching vibration and the stretching vibration of aromatic C=C at 1090 cm^−1^ and 1530 cm^−1^ existed in chitosan, which proved that catechols had reacted with chitosan successfully. Meanwhile, the IR absorption peak at 1300–1090 cm^−1^ was the symmetric vibration caused by the covalent bond between anthraquinone and Fe^2+^, which indicated that the structure of catechol in SDGB had strong reactivity and could produce strong reactivity with metal ions [43,44].

#### 2.2.2. NMR Hydrogen Spectroscopy (HNMR) Analysis

**Figure 2:**
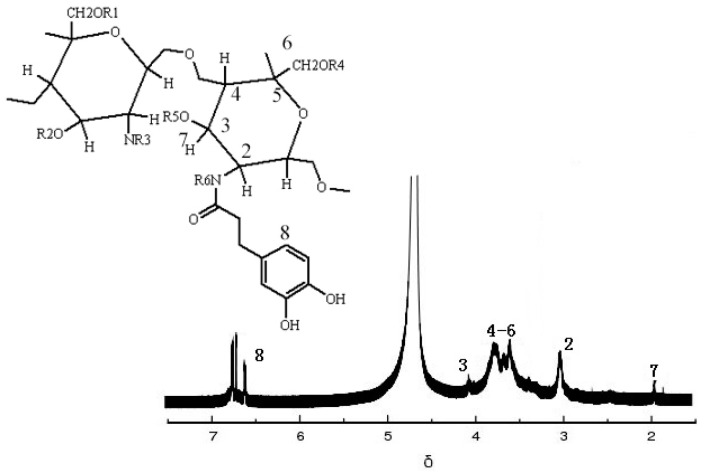
Spectrogram of HNMR.

The HNMR results of SDGB are shown in Figure 2. It can be seen that there is a peak of protons on the main chain of chitosan at chemical displacement δ of 3.6–4.0. The chemical shift δ at 3.1 corresponds to the C-2 proton peak of chitosan, and the chemical shift δ at 1.9 corresponds to the methyl proton peak of acetyl group in chitosan. The peak of chemical shift δ at 6.7 is the proton peak of benzene ring on catechol, and the peak of chemical shift δ at 4.1 is the proton peak of methylene linked to benzene ring, indicating that the polymer containing catechol structure has successfully reacted with chitosan.

#### 2.2.3. Thermogravimetric Analysis

**Figure 3:**
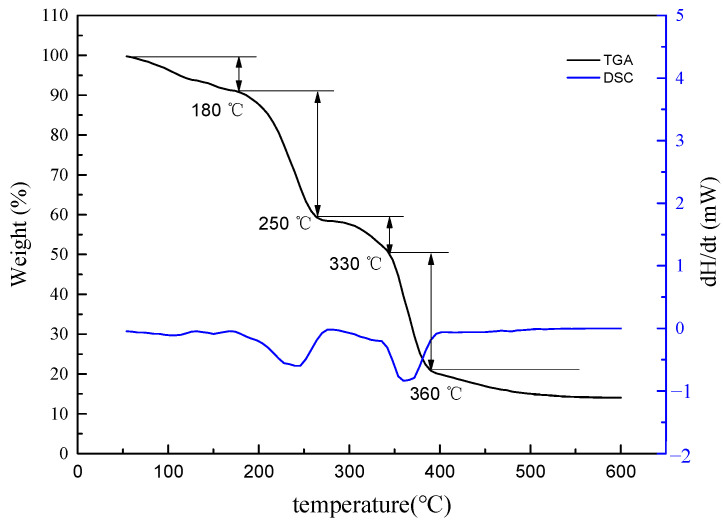
Thermogravimetric analysis results of SDGB.

The thermogravimetric and differential curves of SDGB are shown in Figure 3. The results showed that the weight loss of SDGB could be divided into four stages. From room temperature to about 150 °C, this stage is mainly due to the loss of adsorbed water. From 180 °C to around 245 °C, there is an obvious weight loss at this stage, mainly due to the degradation of the main polymer chains, and the weight loss is about 30%. From 280 °C to 330 °C, this stage was mainly due to the decomposition of side chains and catechol structure. From 330 °C to 360 °C. The chitosan backbone was destroyed and the mass of SDGB remained unchanged after decomposition.

#### 2.2.4. Gel Permeation Chromatography (GPC) Test

The GPC experimental results of SDGB are shown in Table 3. Gel permeation chromatography showed that the number average molar weight of SDGB was 25,974 and the weight average molecular weight was 33,424. The relative molecular weight is moderate, which ensures that it not only has sufficient adsorption strength but also can enter shale microprobes or cracks. Meanwhile, its polydispersity index was low, indicating narrow molecular weight distribution and good homogeneity.

### 2.3. Evaluation of Improving Borehole Stability

#### 2.3.1. Tensile Shear Strength Test

**Figure 4:**
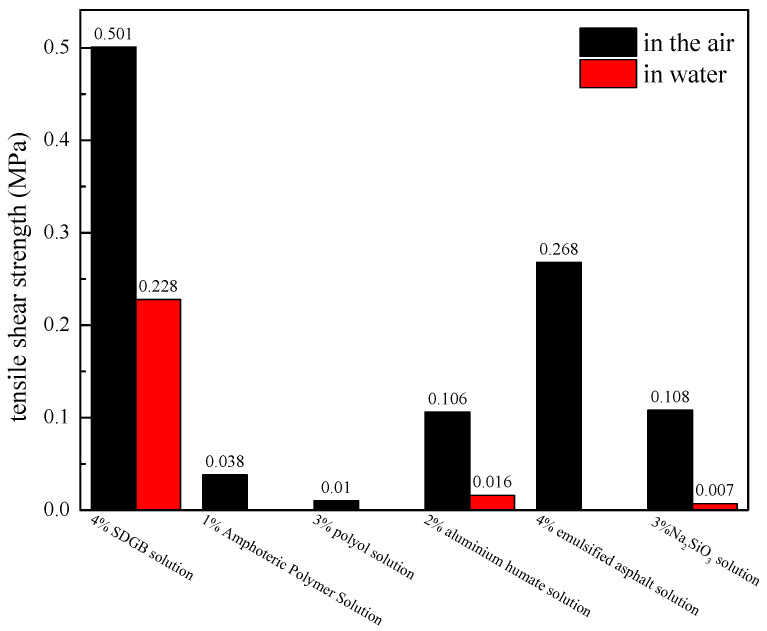
The result of tensile shear strength.

**Figure 5:**
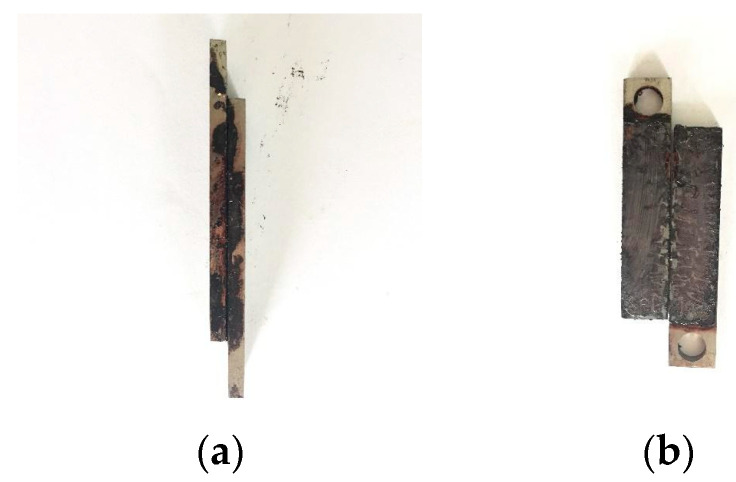
The metal sample treated by 4 wt%SDGB solution. (**a**) Before tested; (**b**) After tested.

The results of the tensile shear strength test of the lapped samples after treated by amphoteric polymer, SDGB, emulsified asphalt, aluminum humate, polyol, and Na_2_SiO_3_ are shown in Figure 4. It can be seen that the lap joint samples treated with SDGB, emulsified asphalt and aluminum humate have higher tensile shear strength in air. The tensile shear strength of 4% SDGB solution in air is about 0.501 MPa, which is the highest among the six samples. The tensile shear strength in water is significantly lower than that in air (0.21 MPa), while the other samples are zero. As can be seen from Figure 5 after treated by SDGB solution, the surface of the metal lap sample is discolored and completely damaged, indicating that SDGB has better adhesion performance. According to Deming TJ et al. [45], catechol group can be oxidized in water to form anthraquinone structure, which can chelate with metal ions or metal oxides on the rock surface to form stable covalent bonds, and then firmly adsorb on the rock surface to prevent rock samples from dispersing in water.

#### 2.3.2. Uniaxial Compressive Strength Test

**Figure 6:**
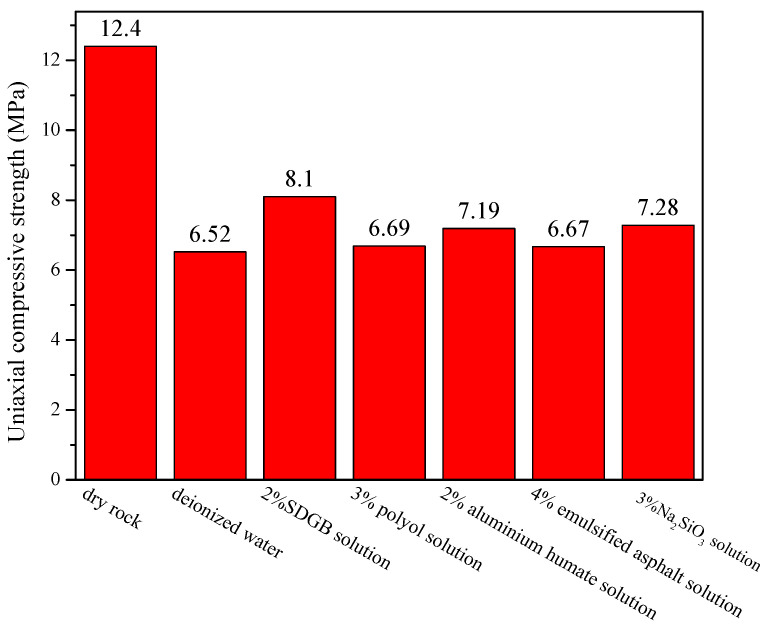
Compressive strength of rock samples treated with different solutions.

The compressive strength test results of rock samples treated with different wellbore stabilizers are shown in Figure 6. The uniaxial compressive strength of dry rock samples is 12.4 MPa, reduced by about 50% after soaking in clean water. After being treated with different wellbore stabilizers, the compressive strength of rock samples is improved. Among them, 2% of SDGB has the best performance. The compressive strength reaches 8.1 MPa, which is about 25% higher than that of clean water. The results show that SDGB can effectively improve the compressive strength of rock samples.

#### 2.3.3. Inhibitory Shale Hydration Test

**Figure 7:**
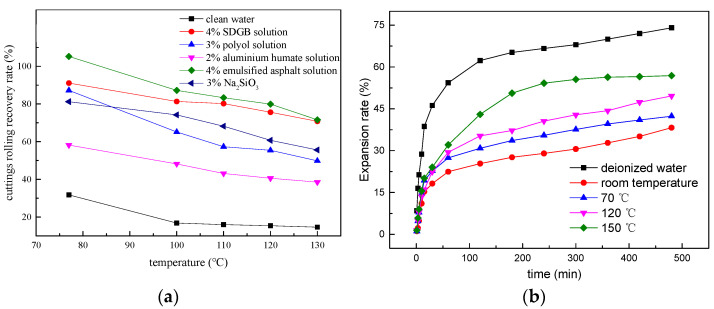
Results of inhibition tests. (**a**) Rolling recovery of different wellbore stabilizers; (**b**) variation of expansion rate at different temperature treated by SDGB.

The main mineral composition of shale used for inhibitive experiments is shown in the Table 4 and Table 5. It can be seen that the clay mineral content of the shale selected for the experiment is 53%. Illte/smectite minerals are the main clay minerals, indicating that the shale used for this experiment has a high content of active minerals and a high potential hydration ability. 2% SDGB solution can effectively inhibit the dispersion of shale cuttings, and the rolling recovery is 91.06% at 70 °C (Figure 7a). With the increase of temperature, the rolling recovery decreases slightly. However, the recovery after 150 °C/16 h hot rolling is still as high as 75.6% (Figure 7b), indicating that SDGB can still effectively inhibit hydration and dispersion at high temperature. The swelling rates of rock samples treated at 70 °C and 150 °C for 8 h are 42% and 57% respectively, which is lower than 74% of fresh water, indicating that the higher the temperature, the worse the inhibition performance.

#### 2.3.4. Hydroscopicity Test

**Figure 8:**
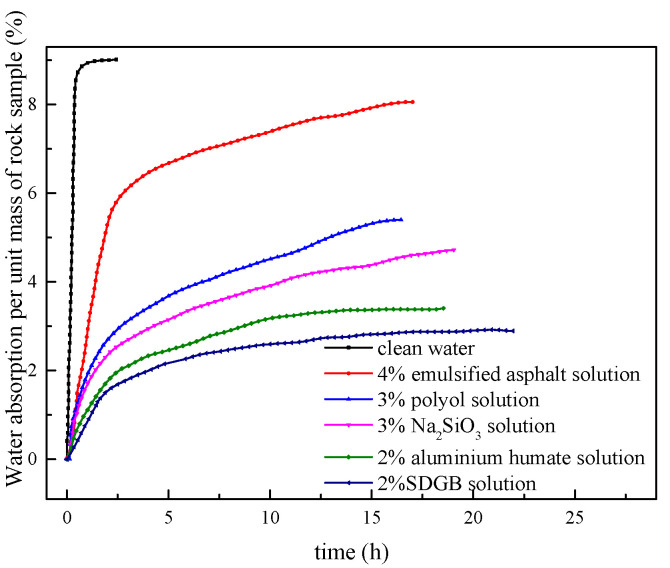
The hydroscopicity curve of different wellbore stabilizers.

The hydroscopicity test results of shale core treated by different wellbore stabilizers are shown in Figure 8 and Table 6. The rock sample has strong water absorption capacity, which tends to be stable in about 2 h, and the total water absorption reaches 9.1%. After treatment with 2% SDGB solution, the total water absorption of rock samples decreased to 2.75%, nearly 70% lower than that of clean water. The results show that SDGB can firmly adsorb and cement rock samples, so as to inhibit the water absorption capacity of rocks and ensure the stability of wellbore.

#### 2.3.5. Compatibility Evaluation in Drilling Fluid

**Figure 9:**
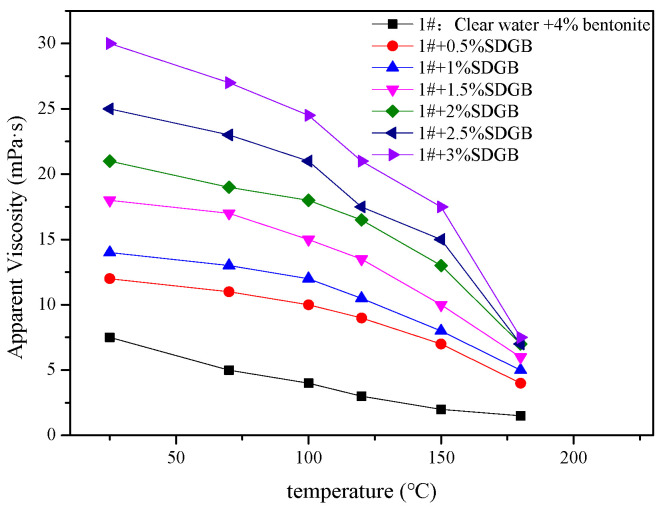
Variation of apparent viscosity of drilling fluid with temperature.

**Figure 10:**
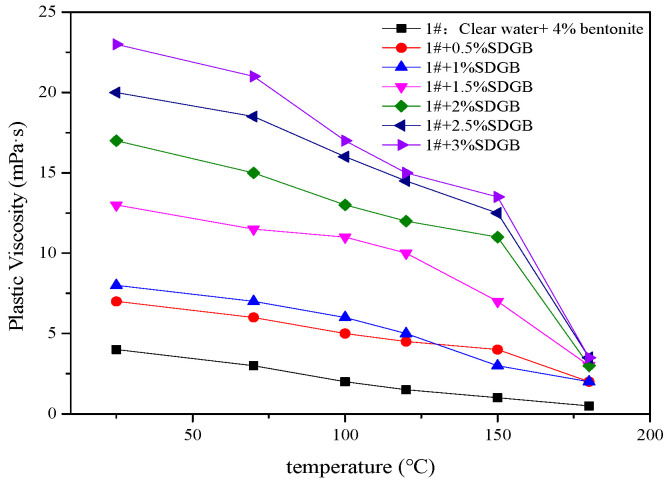
Variation of filtrate loss of drilling fluid with temperature.

**Figure 11:**
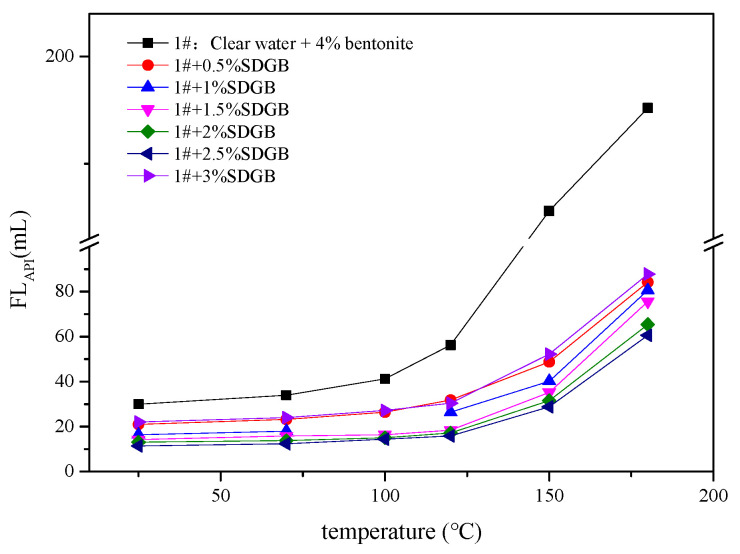
Variation of filtrate loss of drilling fluid with temperature.

Different amounts of SDGB were added to 4 wt% bentonite water-based drilling fluid, and its apparent viscosity, plastic viscosity and filtration were tested after hot rolling at 130 °C/16 h. The results are shown in Figure 9, Figure 10 and Figure 11. It is not difficult to find that there is an optimal concentration of SDGB in the drilling fluid, and the addition of SDGB has no adverse effect on the water-based drilling fluid. After hot rolling at 130 °C, it has good rheological and filtration properties, and has the function of increasing viscosity and reducing filtration.

### 2.4. Mechanism of SDGB

#### 2.4.1. Adsorption Isotherm Test

**Figure 12:**
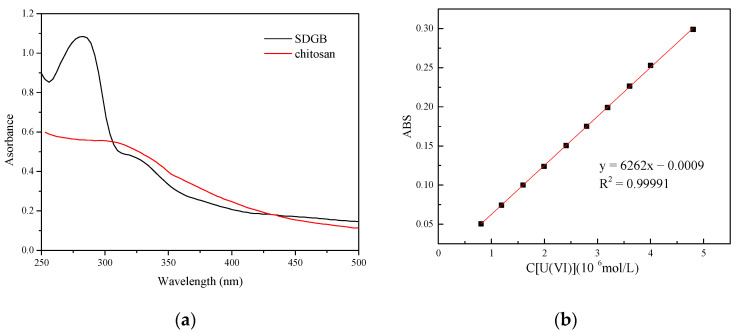
The UV absorption spectra and adsorption standard curve of SDGB. (**a**) UV absorption spectra of SDGB and chitosan; (**b**) SDGB adsorption standard curve.

**Figure 13:**
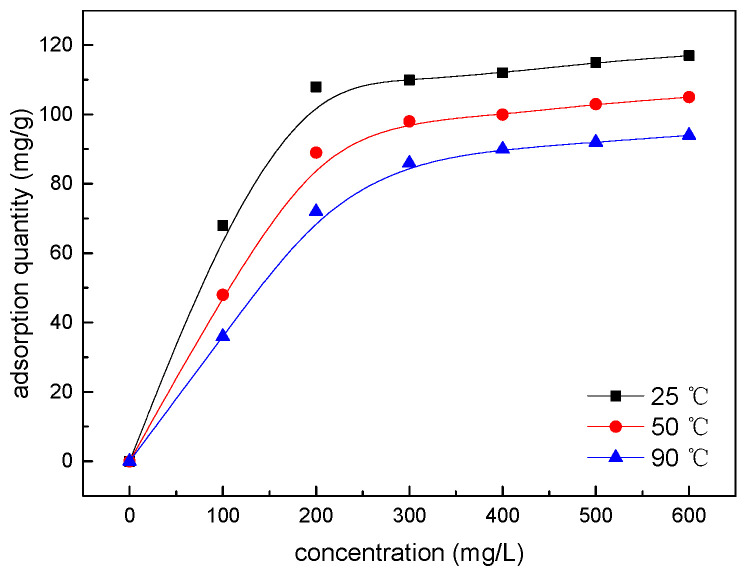
Adsorption isotherm of SDGB.

Figure 12 shows the UV absorption spectrum and adsorption standard curve of SDGB, from which it can be seen that the absorption peak of SDGB is the most obvious at the wavelength of 277.8 nm, and the measured adsorption standard curve is roughly linear. Figure 13 is the curve of sodium adsorption amount of bentonite with different surface temperatures. It shows that the adsorption curves of SDGB at different temperatures are L-shaped, indicating that SDGB mainly adsorbs through a single layer on clay surface. The adsorption capacity of SDGB increases rapidly with increasing concentration. When a certain concentration is reached, the adsorption capacity tends to be flat. The saturated adsorption capacity at 25 °C is 117 mg/g, which shows a strong adsorption capacity. With the increase of temperature, the adsorption capacity of SDGB decreases, and the saturated adsorption capacity can still reach 94 mg/g at 90 °C. SDGB has strong high temperature desorption capacity. The analysis shows that the surface of clay particles is negatively charged and SDGB molecules contain more oxygen-containing functional groups which can be firmly adsorbed on the surface of clay particles by hydrogen bonds.

**Figure 14:**
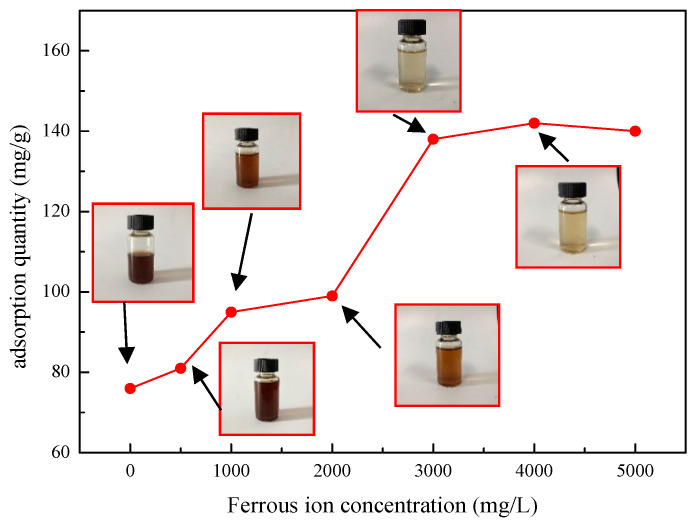
Effect of Fe^2+^ on the adsorption of SDGB.

Shale surfaces are usually rich in metal ions and oxides. Catechol groups can chelate with metal substances to form stable covalent bonds and strongly adsorb on the surface of wellbore rock. In order to further study the adsorption characteristics of SDGB on rock surface, the effects of different concentrations of metal ions (taking ferrous ions as examples) on the adsorption of SDGB were tested. Figure 14 shows the adsorption capacity of SDGB in the presence of Fe^2+^ at different concentrations. The results showed that the adsorption capacity of catechol-chitosan biopolymer capsules on the surface of montmorillonite particles gradually increased with the increase of Fe^2+^ concentration, indicating that the presence of Fe^2+^ can effectively improve the adsorption capacity of catechol-chitosan biopolymer capsules on the surface of montmorillonite particles. According to Deming et al. [45], in the presence of Fe^2+^ ions or other metal ions, oxygen atoms in the catechol structure of SDGB molecules can interact with metal ions to form relatively stable chelation covalent structures, and some can even be oxidized to anthraquinone structures, thereby enhancing the adsorption strength of catechol structures on rock surfaces.

#### 2.4.2. Scanning Electron Microscope Test

**Figure 15:**
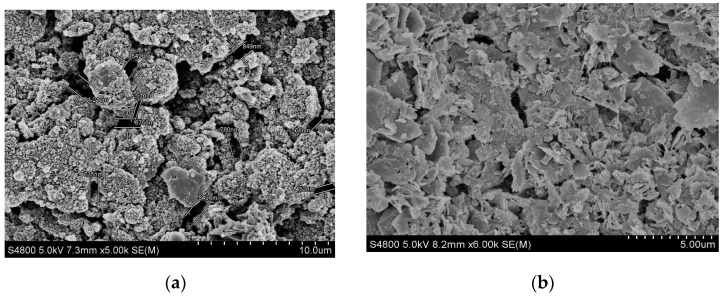
The shale sample treated by SDGB. (**a**) Before treatment (×5.0 k); (**b**) After treatment (×6.0 k).

Figure 15 shows the internal shape of cores before and after different treatments. It can be seen from (a) that micropore and microfissure are well developed in the core and clay minerals adhere to the grain surface. (b) It can be seen that after SDGB treatment, clay minerals originally attached to the grain surface are cemented and filled in the micropore of rock samples, which significantly reduces the micropore in rock samples and makes them more compact. It is found that SDGB not only acts as a seal, but also adsorbs, cements and solidifies clay minerals in micro-cracks of rock samples, thus improving the compressive strength of rock.

#### 2.4.3. FT-IR Test

**Figure 16:**
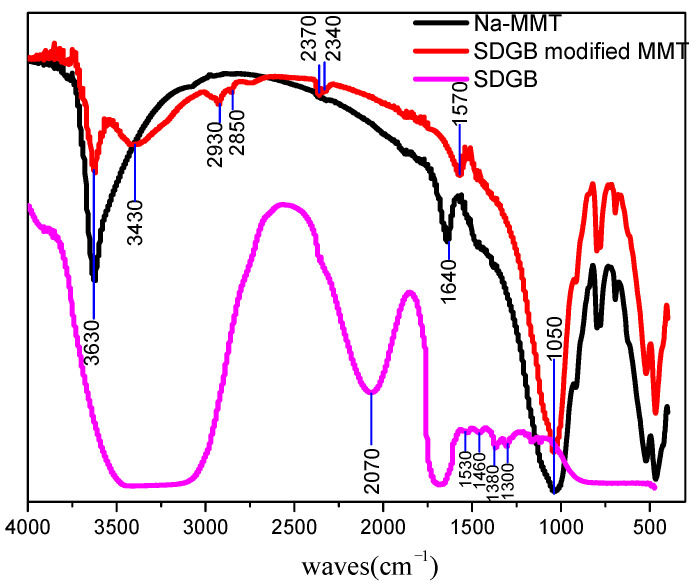
Infrared spectra of sodium montmorillonite before and after modification.

In order to study the interaction mode and mechanism between SDGB and sodium montmorillonite, the infrared spectra of bentonite, SDGB and SDGB modified bentonite were tested, and the result is shown in Figure 16.

It can be seen from the test results that the main absorption peaks of sodium montmorillonite have not changed significantly before and after modification, indicating that the main molecular structure of sodium montmorillonite has not changed before and after SDGB modification. It can be seen from the infrared spectra of Na-MMT that 3630 cm^−1^ can be attributed to the Tensile vibration of the hydroxyl group, because bound water molecules still exist in the molecular layer of Na-MMT, which is the result of the conjugation of freely bound water and bound water. 1050 cm^−1^ is the vibration absorption peak of silica-silicon in sodium montmorillonite molecular layer. 1640 cm^−1^ can be attributed to the tensile vibration of hydroxyl groups adsorbed in water between different layers of sodium montmorillonite. The results showed that the above main absorption peaks did not change significantly before and after modification, which indicated that the action of SDGB and sodium montmorillonite would not change the main molecular structure of bentonite silicate framework. However, some new infrared absorption peaks can also be found in SDGB-modified sodium montmorillonite. 3430 cm^−1^ can be attributed to the tensile vibration absorption peak formed by the near oxygen atoms connected with benzene ring. 2930 cm^−1^ and 2850 cm^−1^ can be attributed to the symmetric vibration absorption peak of methylene. Meanwhile, the intermolecular hydroxy tensile vibration absorption peak initially appeared at 1640 cm^−1^ in the modified Na-MMT spectrum appeared at 1570 cm^−1^ and moved to low frequency to some extent, indicating that SDGB formed hydrogen bond with sodium montmorillonite.

#### 2.4.4. Summary and Analysis of Mechanism

**Figure 17:**
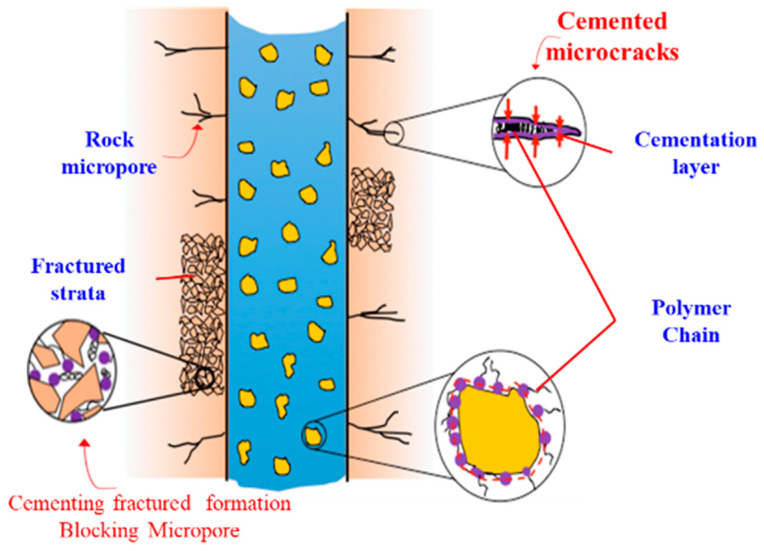
Mechanism diagram of SDGB enhancing wellbore stability.

Traditional high-performance amine shale inhibitors often work through higher amine density. In the presence of a large number of amines, a large number of amines in the inhibitor molecules are positively charged, which can electrostatically absorb with negatively charged bentonite particles, thus neutralizing the negative charges of active clays, compressing their diffusion double layer, driving out water molecules and thus inhibiting shale hydration. The schematic diagram of action mechanism of SDGB is shown in Figure 17. SDGB surface does not contain a large number of strong cationic groups to keep the drilling fluid from functioning (most water-based drilling fluid additives are negatively charged). First, the adsorption of SDGB occurs through the presence of trace metal ions on the shale surface. The structure of catechol in SDGB can chelate with metal ions. The active groups such as catechol adsorb rapidly and firmly on the surface of rock particles on the well wall through hydrogen bond and chelation, encapsulate the active groups, and play a “sticky” role in preventing rock particles from dispersing under the action of drilling fluids and improving rock strength. Second, during the adsorption process between chemical wall fixing agent and the surface of rock particles, the polymer chain can also fill and plug the pores of rock, reduce rock permeability, and improve the stability of drilled rock. Third, the molecules of chemical wall fixing agent contain strong adsorptive groups, which can inhibit hydration when strongly adsorbed on rock surface. In short, the combination of “binder curing to increase strength physical blockage to reduce permeability of rock samples to reduce hydration rejection” improves wellbore stability.

## 3. Conclusions

Inspired by mussels, a novel catechol-conjugated-chitosan biopolymer encapsulators SDGB was synthesized. The optimum synthesis conditions are determined in this paper. The structure of the products was characterized by IR, NMR, and TG. The results showed that the catechol reacted successfully with chitosan, and the decomposition temperature was over 180 °C. The experiment shows that SDGB can effectively improve the shear strength and inhibition of shale. The recovery rate of the shale cuttings was still up to 75.6% after 150 °C/16 h hot rolling, and the bearing capacity of the rock can be increased by 24%. A wellbore strengthening drilling fluid was constructed based on SDGB. The rheological and filtration performance of this drilling fluid is stable before and after 130 °C/16 h hot rolling, and shale inhibition behavior is good. Moreover, SDGB could chelate with metal ions, forming a stable covalent bond, which plays an important role in adhesiveness, inhibition, and blockage.

## 4. Materials and Methods

### 4.1. Materials

3,4-dihydroxybenzoic acid(catechol), chitosan (degree of deacetylation (>95%), *N*-(3-dimethylaminopropyl)-*N*-ethylcarbodiimide hydrochloride (EDC) and *N*-hydroxysuccinimide (NHS) were commercial products from Aladdin reagent company, Shanghai, China, respectively. Na_2_SO_3_, NaCl, CaCl_2_, NaOH, and Na_2_SiO_3_ were purchased from China Pharmaceutical Reagents Co., Ltd. in Shanghai, China. Sodium-based bentonite and artificial cores for drilling fluid were purchased from Huawei Bentonite Group Co., Ltd. in Weifang City, China and Haian Petroleum Research Instrument Co., Ltd. in Nantong City, China, respectively. The main reagents for the reaction are detailed in Table 4.

### 4.2. Methods

#### 4.2.1. Synthesis of Catechol-Chitosan Biopolymer Encapsulators

**Figure 18:**
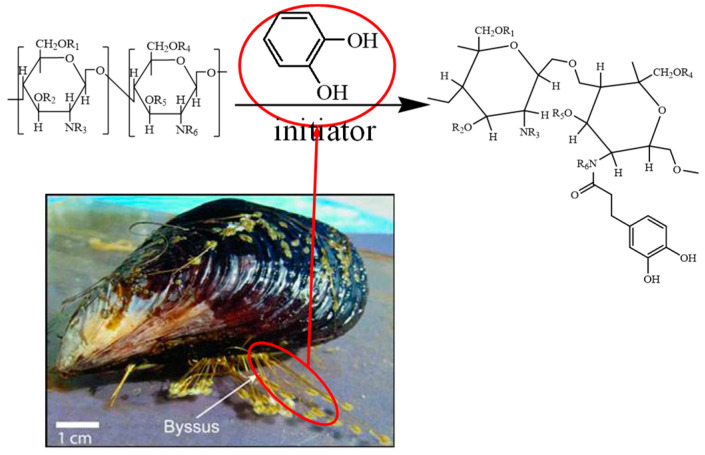
The molecular structure of SDGB.

Ferrous chloride was added and fully dissolved in hot ethanol solution (50% wt, 30 mL). A certain amount of polymer containing catechol structure was added into a three-neck flask and stirred with an electric stir bar under an inert N_2_ atmosphere. A certain amount of chitosan was dissolved with 20 mL, 1 mol/L hydrochloric acid and them nitrogen is introduced into the reaction system. After the pH was adjusted to a certain value with 5 mol/L NaOH solution, the initiator is dissolved in 10 mL of deionized water and then added dropwise to the reaction solution to trigger the reaction. The mixture was refluxed for a period of time at a certain temperature. The precipitate was filtered and washed with ethanol to get the final product. The molecular structure of SDGB is shown in Figure 18.

Orthogonal tests were used to optimize the four main factors to determine the best formulation and these four factors are the mole ratio between catechol and chitosan, reaction time, initiator concentration, and reaction temperature. The influence of different factors was analyzed by testing the tensile shear strength and rolling recovery of the product.

#### 4.2.2. Tensile Shear Strength Test

**Figure 19:**
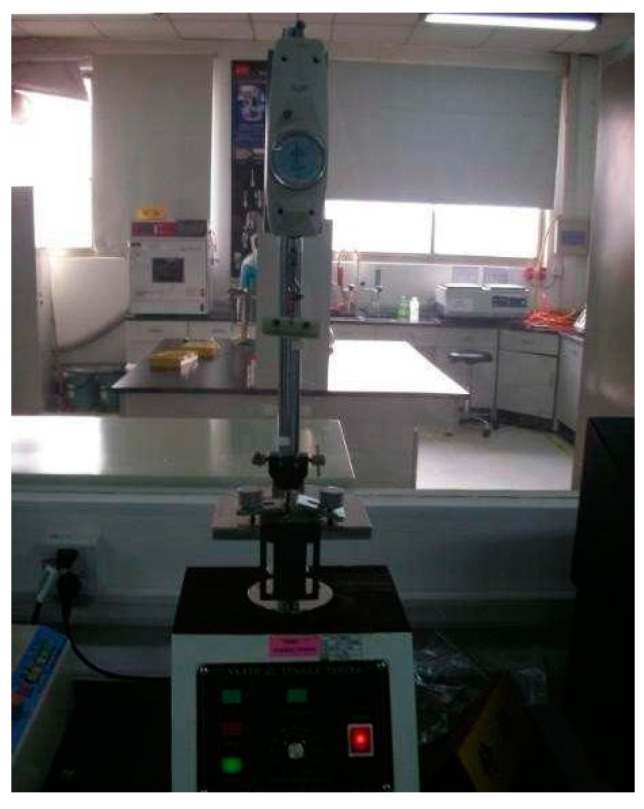
WDW-100 tensile shear strength device.

The mechanical properties of core before and after treated by SDGB are tested by uniaxial mechanical strength test(Bairoe Test Instrument Co., Ltd. in Shanghai, China), which can effectively reflect the improvement of shale by strong wall drilling fluids. In accordance with the Chinese National standard “methods for determination of tensile shear strength of adhesives” (GB7124-1986) and “determination of chemical resistance of adhesives” (GB/t13353-92), the aqueous solution of SDGB is applied to the single lap surface of the sample and the lap joint sample is pressed at 5 MPa for 2 h, then placed in water at 50 °C for 24 h. Then longitudinal tensile force is applied to the single lap joint surface of the sample to test the maximum load that the sample can bear on wdw-100 microcomputer controlled triaxial multifunctional machine (Figure 19).

#### 4.2.3. Cuttings Hot-Rolling Dispersion Test

Shale dispersion recovery experiment is a common method to evaluate the shale inhibition ability of treatment agent. The dispersion performance of shale is related to the stability of wellbore. It is one of the important indexes to evaluate the stability of shale wellbore macroscopically. The dispersion experiment can be used to understand the hydration and dispersion performance of rock samples, and can also be used as a means to evaluate the inhibition of drilling fluid on shale. 350 mL of solution with encapsulators of various concentrations and 50 g of shale cuttings (2–5 mm) obtained from the upper layer of Shahejie formation in Dongying oil field were added into sealed cells. After hot rolling at 77 °C for 16 h, the cuttings were washed with 10% KCl solution and screened through a 40-mesh sieve. The recovered cuttings were dried at 105 °C for 4 h and the percentage of recovery was determined.

#### 4.2.4. Characterization of SDGB

A Bruker AVANCE 400 NMR spectrometer (Brooke Co., Ltd., Zurich, Switzerland) was used to measure the proton nuclear magnetic resonance (^1^HNMR) spectra. D_2_O was used for field frequency lock, and the observed ^1^H chemical shifts were reported in parts per million (ppm). Before ^1^H NMR analysis, the pH value of the polymer solution was adjusted to around 9.0 by dilute NaOH/D_2_O solution.

A total of 1 mg dry SDGB powder and 20 mg KBr were mixed fully. The mixture was loaded into the mold and compacted with 50 MPa pressure. FTIR of the compacted tableting were obtained on a NEXUS FT-IR spectrometer(Thermo Nicolet Corporation, Waltham, MA, USA).

The thermogravimetric analysis of SDGB was carried out by mettler-teledothermo gravimetric analyzer (NETZSCH-Gerätebau Co., Ltd., Selb, Germany). The temperature range was from room temperature to 1000 °C, the heating rate was 10 K/min, and the atmosphere was nitrogen, and the gas flow rate was 50 mL/min.

The relative molecular mass of SDGB was determined by the SFD gel permeation chromatograph (GPC) (Schambeck Co., Ltd., Berlin, Germany). The mobile phase was phosphate buffer solution. The column was SHODEX (K-806M chloroform system, and the filler was styrene and two vinyl benzene copolymer).

#### 4.2.5. Core Uniaxial Compressive Strength Test

The rock samples were soaked in different wellbore strengthening agent solutions for 4 h, then taken out carefully and dried at room temperature. Then compressive strength was tested on wdw-100 microcomputer controlled triaxial multifunctional machine (Bairoe Test Instrument Co., Ltd., Shanghai, China).

#### 4.2.6. The Hydroscopicity Test

The hydroscopicity test was conducted by self-made core self-priming experimental device. The water absorption and water absorption rate of typical shale in deionized water were tested. At the same time, the change of water absorption of rock samples treated with different encapsulators with time was tested to comprehensively evaluate the hydration and dispersion performance of rock samples. First, the experimental core is dried in an oven at 105 °C for 12 h, and then placed at 25 °C for 24 h. During the experiment, the room temperature is maintained at 25 °C. Then, the shale standard core is suspended on the hook of the electronic balance, so that its lower end just contacts the solution. The total mass of the rock sample changes after water absorption over time is recorded.

#### 4.2.7. Scanning Electron Microscope Test

Core micro morphology was tested by S-4800 field emission scanning electron microscope (HitaChi, Ltd., Tokyo, Japan) after being treated by SDGB, sodium silicate and aluminum humate.

#### 4.2.8. Adsorption Isotherm Test

In order to further study the action mechanism of SDGB in drilling fluids, the adsorption characteristics of SDGB on bentonite particles were studied.

(1). A sufficient amount of 10% hydrogen peroxide was added to the beaker together with 100 g of bentonite and stirred simultaneously on a heating oven to eliminate the possible influence of the residual organics in the bentonite on the adsorption test results. Deionized water was then added so that the contents in the beaker are well dispersed, and then was placed in a centrifuge and centrifuged at 8000 rpm for 15 min. The upper clear liquid was poured out, and deionized water was continued to be centrifuged repeatedly for three times. The precipitate was collected and then placed into a blast drying oven and dried to constant weight, crushed and sieved.

(2). Some SDGB was added into a beaker containing deionized water, stirred well and dissolved; 2.0 g of purified bentonite was added to another beaker containing deionized water, stirred well and dispersed. After the two beakers had been mixed, they were placed in a magnetic stirrer and stirred thoroughly for 24 h until the adsorption equilibrium was reached. The mixed suspension was placed in a centrifuge and centrifuged at a high speed of 10,000 rpm for 15 min. The absorbance in λ = 200–400 nm of the supernatant was measured by a uv-1750 UV VIS spectrophotometer(Yuanxi Instrument Co., Ltd., Shanghai, China).

(3). In λ = 277.8 nm, the adsorption capacity on the surface of bentonite sodium was calculated from the standard curve of polymer solution concentration and light transmittance (as shown in Figure 12a).

#### 4.2.9. Rheological and Filtration Testing of Drilling Fluids

(1) Drilling fluid preparation and hot rolling

A total of 16 g of sodium-based bentonite was added to 400 mL of clear water, and stirred at 8000 rpm in a high-speed blender for 30 min. Then 0.8 g of Na_2_CO_3_ was added, stirred for 20 min, and then pre-hydrated for 24 h. A certain amount of polymer was then added to the slurry, which was stirred at 8000 rpm for 30 min in a high-speed blender. The composites were aged at set temperature for 16 h by hot rolling and were cooled to room temperature before stirring at high speed for 20 min. Rheological and filtration properties of drilling fluid before and after rolling at a specific temperature/16 h were tested according to the drilling fluid performance evaluation standard SY/t5621-1993.16g.

(2) API Static Filtration Test

The static API filterability of drilling fluid was tested with ZNZ-D3 API medium pressure filter (Qingdao Haitong Instrument Co., Ltd., Qingdao, China). A certain amount of drilling fluid was loaded into the filter kettle and the top was covered with API filter paper and placed under a pressure of 100 psi. The filtered volume (FLAPI) of the drilling fluid is recorded for 30 min, which is recommended by API.

(3). Rheological property test

The rheological parameters of the drilling fluid are tested according to the drilling fluid performance evaluation standard SY/T5621-1993. Apparent viscosity, plastic viscosity, and shear force of drilling fluid were measured with ZNP-M7 6-speed rotating viscometer (Haitong Instrument Co., Ltd., Qingdao, China). Then the apparent viscosity, plastic viscosity, and shear force of drilling fluid with φ600 and φ were measured. The value of 300 is calculated according to the test program recommended by API.

AV = φ600/2(1)

PV = φ600 − φ300(2)

where:

AV is the apparent viscosity (mPa·s);

PV is the plastic viscosity (mPa·s);

φ600 is the dial reading of 6-speed rotational viscometer at 600 r/min (dia);

φ300 is the dial reading of 6-speed rotational viscometer at 300 r/min (dia).

## Figures and Tables

**Table 1:** Orthogonal tests with three levels and four factor designs.

Factor Level	Mole Ratio of Catechol/Chitosan	Initiator Concentration * /%	Time /h	Temperature /°C
1	1:1	5	8	10
2	1:2	7	10	25
3	1:3	10	12	40

* Initiator amount is the weight percentage of initiator in total monomer.

**Table 2:** Optimized results of SDGB orthogonal experiments.

Num.		Monomer Ratio	Initiator Concentration /%	Time /h	Temperature /°C	Rolling Recovery /%
1		1:1	5	8	10	75.1
2		1:1	7	10	25	73.6
3		1:1	10	12	40	67.5
4		1:2	5	10	40	74.6
5		1:2	7	12	10	78.9
6		1:2	10	8	25	75.1
7		1:3	5	12	25	73.4
8		1:3	7	8	40	74.3
9		1:3	10	10	10	78.4
Level	k1	0.721	0.744	0.748	0.775	-
	k2	0.762	0.756	0.755	0.74	-
	k3	0.754	0.737	0.733	0.721	-
R		0.041	0.019	0.023	0.053	-

**Table 3:** Relative molecular weight test results of SDGB.

Mn (Dalton)	Mw (Dalton)	Mp (Dalton)	Mz (Dalton)	Mz_+1_ (Dalton)	Polydispersity Index
25,974	33,424	17,339	46,291	66,870	1.66731

**Table 4:** The main mineral composition of shale used for inhibitive experiments.

Quartz	Plagioclase	Calcite	Hematite	Clay Mineral
32	8	2	2	53

**Table 5:** The main clay composition of shale used for inhibitive experiments.

Kaolinite	Chlorite	Illite	Illte/Smectite	Interlayer Ratio (%)
1	0	2	97	75

**Table 6:** The hydroscopicity result of different wellbore stabilizers.

Wellbore Stabilizer	Water Absorption of Rock Core (%)	Water Absorption Reduction Rate (%)
Clean water	9.10	0
4% emulsified asphalt solution	8.05	11.53
3% polyol solution	5.38	40.88
3% Na_2_SiO_3_ solution	4.70	48.35
2% aluminium humate solution	3.24	64.40
2% SDGB solution	2.75	69.78
Clean water	9.10	0
4% emulsified asphalt solution	8.05	11.53

## References

[1] Lee H., Ong S.H., Azeemuddin M., Goodman H. (2012). A wellbore stability model for formations with anisotropic rock strengths. J. Pet. Sci. Eng..

[2] Huang X., Shen H., Sun J., Lv K., Liu J., Dong X., Luo S. (2018). Nanoscale laponite as a potential shale inhibitor in water-based drilling fluid for stabilization of wellbore stability and mechanism study. ACS Appl. Mater. Interfaces.

[3] Bauder T., Barbarick K., Ippolito J., Shanahan J., Ayers P. (2005). Soil properties affecting wheat yields following drilling-fluid application. J. Environ. Qual..

[4] Tianshou M., Ping C. (2014). Study of meso-damage characteristics of shale hydration based on CT scanning technology. Pet. Explor. Dev..

[5] Bird P. (1984). Hydration-phase diagrams and friction of montmorillonite under laboratory and geologic conditions, with implications for shale compaction, slope stability, and strength of fault gouge. Tectonophysics.

[6] Heidug W., Wong S.W. (1996). Hydration swelling of water-absorbing rocks: A constitutive model. Int. J. Numer. Anal. Methods Geomech..

[7] Shadizadeh S.R., Moslemizadeh A., Dezaki A.S. (2015). A novel nonionic surfactant for inhibiting shale hydration. Appl. Clay Sci..

[8] Liu H., Meng Y., Li G., Li P., Deng Y. (2010). Theoretical simulation and experimental evaluation of the effect of hydration on the shale rock strength. Drill. Prod. Technol..

[9] Zhong H., Qiu Z., Tang Z., Zhang X., Zhang D., Huang W. (2016). Minimization shale hydration with the combination of hydroxyl-terminated PAMAM dendrimers and KCl. J. Mater. Sci..

[10] Gou S., Yin T., Liu K., Guo Q. (2015). Water-soluble complexes of an acrylamide copolymer and ionic liquids for inhibiting shale hydration. New J. Chem..

[11] Zeng F., Zhang Q., Guo J., Zeng B., Zhang Y., He S. (2021). Mechanisms of shale hydration and water block removal. Pet. Explor. Dev..

[12] Simpson J., Walker T., Jiang G. (1995). Environmentally acceptable water-base mud can prevent shale hydration and maintain borehole stability. SPE Drill. Completion.

[13] Liu Y., Chen L., Tang Y., Zhang X., Qiu Z. (2022). Synthesis and characterization of nano-SiO_2_@ octadecylbisimidazoline quaternary ammonium salt used as acidizing corrosion inhibitor. Rev. Adv. Mater. Sci..

[14] Baoyou R., Xiaolin P., Cheng C., Chuan M. (2018). Experimental study on improving the formation pressure-bearing capacity by using nano-drilling fluid. Oil Drill. Prod. Technol..

[15] Kang Y., Xu C., Tang L., Li S., Li D. (2014). Constructing a tough shield around the wellbore: Theory and method for lost-circulation control. Pet. Explor. Dev..

[16] Li J., Qiu Z., Liu Z., Yang Y., Song H., Liang Y. Application of wellbore strengthening drilling fluid technology in Lingshui gas field. Proceedings of the IOP Conference Series: Earth and Environmental Science.

[17] Qiu Z., Bao D., Li J., Liu J., Chen J. (2018). Mechanisms of wellbore strengthening and new advances in lost circulation control with dense pressure bearing zone. Drill. Fluid Completion Fluid.

[18] Huang X., Sun J., Lv K., Liu J., Shen H., Zhang F. (2018). Application of core-shell structural acrylic resin/nano-SiO_2_ composite in water based drilling fluid to plug shale pores. J. Nat. Gas Sci. Eng..

[19] Hoxha B.B., van Oort E., Daigle H. (2019). How do nanoparticles stabilize shale?. SPE Drill. Completion.

[20] Akhtarmanesh S., Shahrabi M.A., Atashnezhad A. (2013). Improvement of wellbore stability in shale using nanoparticles. J. Pet. Sci. Eng..

[21] Zhong H., Gao X., Zhang X., Qiu Z., Zhao C., Zhang X., Jin J. (2020). Improving the shale stability with nano-silica grafted with hyperbranched polyethyleneimine in water-based drilling fluid. J. Nat. Gas Sci. Eng..

[22] Ghasemi A., Jalalifar H., Apourvari S.N., Sakebi M.R. (2019). Mechanistic study of improvement of wellbore stability in shale formations using a natural inhibitor. J. Pet. Sci. Eng..

[23] Pourkhalil H., Nakhaee A. (2019). Effect of nano ZnO on wellbore stability in shale: An experimental investigation. J. Pet. Sci. Eng..

[24] Zhang J., Li L., Wang S., Wang J., Yang H., Zhao Z., Zhu J., Zhang Z. Novel micro and nano particle-based drilling fluids: Pioneering approach to overcome the borehole instability problem in shale formations. Proceedings of the SPE Asia Pacific Unconventional Resources Conference and Exhibition.

[25] Jung C.M., Zhang R., Chenevert M., Sharma M. High-performance water-based mud using nanoparticles for shale reservoirs. Proceedings of the SPE/AAPG/SEG Unconventional Resources Technology Conference.

[26] Luo Z., Wang L., Yu P., Chen Z. (2017). Experimental study on the application of an ionic liquid as a shale inhibitor and inhibitive mechanism. Appl. Clay Sci..

[27] Zhong H., Qiu Z., Huang W., Cao J. (2012). Poly (oxypropylene)-amidoamine modified bentonite as potential shale inhibitor in water-based drilling fluids. Appl. Clay Sci..

[28] Huang X., Sun J., Jin J., Lv K., Li H., Rong K., Zhang C., Meng X. (2021). Use of silicone quaternary ammonium salt for hydrophobic surface modification to inhibit shale hydration and mechanism study. J. Mol. Liq..

[29] Zhang S., He Y., Chen Z., Sheng J.J., Fu L. (2018). Application of polyether amine, poly alcohol or KCl to maintain the stability of shales containing Na-smectite and Ca-smectiteShifeng Zhang et al. Maintaining stability of shale with Na-, Ca-smectite. Clay Miner..

[30] Patel A.D. Design and development of quaternary amine compounds: Shale inhibition with improved environmental profile. Proceedings of the SPE International Symposium on Oilfield Chemistry.

[31] Ali J.A., Hamadamin A.B., Ahmed S.M., Mahmood B.S., Sajadi S.M., Manshad A.K. (2022). Synergistic Effect of Nanoinhibitive Drilling Fluid on the Shale Swelling Performance at High Temperature and High Pressure. Energy Fuels.

[32] Schlemmer R.F. (2007). Membrane Forming In-Situ Polymerization for Water Based Drilling Fluids. U.S. Patent.

[33] Schlemmer R., Friedheim J., Growcock F., Bloys J., Headley J., Polnaszek S. (2003). Chemical osmosis, shale, and drilling fluids. SPE Drill. Completion.

[34] Schlemmer R., Friedheim J., Growcock F., Bloys J., Headley J., Polnaszek S. Membrane efficiency in shale-an empirical evaluation of drilling fluid chemistries and implications for fluid design. Proceedings of the IADC/SPE Drilling Conference.

[35] Albooyeh M., Kivi I.R., Ameri M. (2018). Promoting wellbore stability in active shale formations by water-based muds: A case study in Pabdeh shale, Southwestern Iran. J. Nat. Gas Sci. Eng..

[36] Chu Q., Luo P., Zhao Q., Feng J., Kuang X., Wang D. (2013). Application of a new family of organosilicon quadripolymer as a fluid loss additive for drilling fluid at high temperature. J. Appl. Polym. Sci..

[37] Fang W., Jiang H., Li J., Li W., Li J., Zhao L., Feng X. (2016). A new experimental methodology to investigate formation damage in clay-bearing reservoirs. J. Pet. Sci. Eng..

[38] Gomez S., He W. Fighting wellbore instability: Customizing drilling fluids based on laboratory studies of shale-fluid interactions. Proceedings of the IADC/SPE Asia Pacific Drilling Technology Conference and Exhibition.

[39] Tan C.P., Richards B.G., Rahman S. Managing physico-chemical wellbore instability in shales with the chemical potential mechanism. Proceedings of the SPE Asia Pacific Oil and Gas Conference.

[40] Zhang Q., Jia W., Fan X., Liang Y., Yang Y. (2015). A review of the shale wellbore stability mechanism based on mechanical–chemical coupling theories. Petroleum.

[41] Kord Forooshani P., Lee B.P. (2017). Recent approaches in designing bioadhesive materials inspired by mussel adhesive protein. J. Polym. Sci. Part A Polym. Chem..

[42] Lee H., Rho J., Messersmith P.B. (2009). Facile conjugation of biomolecules onto surfaces via mussel adhesive protein inspired coatings. Adv. Mater..

[43] Guo Z., Ni K., Wei D., Ren Y. (2015). Fe^3+^-induced oxidation and coordination cross-linking in catechol–chitosan hydrogels under acidic pH conditions. RSC Adv..

[44] Shu L., Chiou Y.-M., Orville A.M., Miller M.A., Lipscomb J.D., Que L. (1995). X-ray absorption spectroscopic studies of the Fe (II) active site of catechol 2,3-dioxygenase. Implications for the extradiol cleavage mechanism. Biochemistry.

[45] Deming T.J. (1999). Mussel byssus and biomolecular materials. Curr. Opin. Chem. Biol..

